# Angiotensin-Converting Enzyme Inhibitors and Other Medications Associated With Angioedema

**DOI:** 10.7759/cureus.49306

**Published:** 2023-11-23

**Authors:** Layne Landry, Taylor Witten, Ahmed I Anwar, Corrie N Jackson, Norris C Talbot, Shahab Ahmadzadeh, Giustino Varrassi, Sahar Shekoohi, Alan D Kaye

**Affiliations:** 1 School of Medicine, Louisiana State University Health Sciences Center, Shreveport, USA; 2 Psychology, Quinnipiac University, Hamden, USA; 3 Anesthesiology, Louisiana State University Health Sciences Center, Shreveport, USA; 4 Pain Medicine, Paolo Procacci Foundation, Rome, ITA

**Keywords:** nonsteroidal anti-inflammatory drugs (nsaids), bradykinin, pathophysiology, adverse drug reactions, drug-induced angioedema, ace inhibitor, angioedema

## Abstract

Angioedema is a localized swelling of the dermis, subcutaneous tissues, and/or submucosal tissues caused by fluid extravasation into these tissues. Angioedema is associated with certain vasoactive molecules and is typically mediated by histamine or bradykinin. It manifests clinically as facial edema, swelling of the extremities and urogenital area, and potential involvement of the larynx, leading to dyspnea and inspiratory stridor, which can become life-threatening. Histamine-mediated angioedema is associated with urticaria and pruritus and will show classic signs of allergic (type 1 hypersensitivity) reactions. Bradykinin-mediated angioedema is often familial (hereditary angioedema) and is more often associated with gastrointestinal symptoms (abdominal pain, nausea, vomiting, diarrhea), edema of the extremities and trunk, and a lack of urticaria and pruritus. Angiotensin-converting enzyme inhibitors (ACEIs) are a class of medications commonly prescribed for hypertension, heart failure, and diabetic nephropathy. ACEIs are associated with an increased risk of angioedema, which can range from a mild reaction to severe and life-threatening. ACEI-induced angioedema is a bradykinin-mediated reaction that can occur in individuals with a genetic predisposition. Other medications, such as angiotensin receptor blockers, nonsteroidal anti-inflammatory drugs, and certain antibiotics, most notably those in the beta-lactam class, can also cause drug-induced angioedema. The present investigation describes current knowledge of the pathophysiology, epidemiology, clinical manifestations, predisposing factors, and management of drug-induced angioedema.

## Introduction and background

Pathophysiology

Drug-induced angioedema via mechanisms mediated by the accumulation of vasoactive molecules, most notably histamine, bradykinin, and leukotrienes [1]. Histamine-mediated angioedema (allergic) is caused by degranulation of mast cells and histamine release via an IgE-mediated mechanism most commonly associated with beta-lactam antibiotics as well as non-steroidal anti-inflammatory drugs (NSAIDs), usually aspirin [1,2]. Bradykinin-mediated angioedema (non-allergic) is associated with HAE and angiotensin-converting enzyme inhibitor (ACEI)-induced angioedema and is caused by a decrease in bradykinin degradation, leading to accumulation and subsequent extravasation of fluid causing symptoms [1]. Leukotriene-mediated angioedema occurs mostly with NSAIDs and is due to cyclooxygenase-1 inhibition leading to shunting of arachidonic acid toward the production of leukotriene B4, C4, and D4, ultimately causing vasodilation and angioedema [3]. In this regard, NSAIDs can cause either histamine-mediated or leukotriene-mediated angioedema.

Epidemiology

Angioedema is responsible for 80,000-112,000 emergency department (ED) visits yearly, with a hospitalization rate of 4 per 100,000 [4-6]. Histamine-mediated forms account for 40-70% of all cases of angioedema [6,7]. ACEI-induced angioedema accounts for 30% of angioedema cases presenting to the ED [6]. NSAID-induced angioedema occurs in approximately 0.1-0.3% of patients using NSAIDs [8]. In patients taking ACEIs, the lifetime incidence of angioedema ranges from 0.1% to 0.7% [6]. Additionally, 1.2-5.1% of patients receiving treatment with tissue plasminogen activator (tPA) after a stroke will experience angioedema, and the risk is increased if the patient is taking an ACEI [6,9].

Genetic and environmental factors

Several genes have been studied and found to be associated with ACEI-induced angioedema. One study found that a single nucleotide polymorphism (C-2399A) in the XPNPEP2 gene related to lower activity of aminopeptidase P, one of the primary inactivators of bradykinin, was associated with ACEI-induced angioedema in men only [10]. Another study found that a polymorphism in the gene encoding neprilysin (MME, rs989692), which is responsible for the breakdown of substance P and bradykinin, was significantly associated with ACEI-induced angioedema in African Americans [11]. Mutations involving steps in the arachidonic acid pathway have been shown to play a role in the development of NSAID-induced urticaria/angioedema [3,12].

ACEI-induced angioedema is significantly more common in African Americans, with studies showing a 4.5-5 times greater incidence when compared to Caucasians [1,13]. Personal history of allergies or use of antihistamines has been shown to increase the risk of ACEI-induced angioedema, as well as some studies showing a seasonal correlation in the number of cases reported [14-16]. Those taking immunosuppressants or dipeptidyl peptidase IV (DPP-IV) inhibitors along with an ACEI are at increased risk of developing drug-induced angioedema [6,17,18].

Clinical manifestations

Bradykinin-mediated angioedema (ACEI-induced) typically involves edema of the lips, tongue, and face but can also include the larynx, extremities, trunk, and bowel [1]. The location of edema in these patients usually displays no specific pattern and can involve multiple sites without continuous distribution [6]. The bradykinin-mediated form has a slower onset of hours and will last between 48 and 72 hours [6]. The involvement of the gastrointestinal tract usually occurs in patients with hereditary angioedema (HAE) and can mimic the symptoms of bowel obstruction [19]. Histamine-mediated angioedema (NSAID-induced, beta-lactam-induced, etc.) more commonly involves anaphylaxis-type symptoms such as urticaria, hypotension, and bronchospasm/wheezing compared to the bradykinin-mediated mechanism [6]. The histamine-mediated form usually has a faster onset of minutes and lasts between 12 and 24 hours [6]. Urticaria is rare in the non-histaminergic forms of angioedema but occurs in around half of all patients with the histamine-mediated form [17,20].

Management

The first step in the management of angioedema is to evaluate the patient for signs of anaphylaxis, a common mimic of angioedema, and assess for airway compromise. If the patient presents with stridor, hoarseness, dyspnea, and voice changes, a definitive airway may be required [6,21]. The next most important step in treatment, once anaphylaxis has been ruled out, is to remove the offending agent that is likely causing the symptoms [6]. Patients who likely have a histaminergic form of angioedema can be treated with epinephrine (initial dose of 0.3-0.5 milliliters of 1:1000 dilution via intramuscular injection repeated every 5-20 minutes as needed), steroids (methylprednisolone 125 milligrams intravenously), antihistamines (diphenhydramine 25-50 milligrams intravenously in combination with a second or third generation antihistamine agent such as cetirizine or loratadine), and intravenous fluids [6]. If repeated doses of intramuscular epinephrine are required, then intravenous epinephrine should be considered at a dose of 1-4 micrograms per minute [6]. These measures will be insufficient for patients with bradykinin-mediated angioedema; however, these patients will require treatment with medications such as C1 esterase inhibitor concentrates, kallikrein inhibitors, and bradykinin-2-receptor antagonists [6,22,23]. Additionally, case reports have shown that fresh frozen plasma (FFP) can lead to improvement in HAE and ACEI-induced angioedema due to C1-INH contained therein [24-26].

## Review

Angiotensin-converting enzyme inhibitors

Angioedema is the extravasation of fluid into the interstitial space and surrounding tissues because of increased vascular permeability and vasodilation [27]. This swelling can be classified as either hereditary, allergy-induced, or drug-induced, depending on the inflammatory mediator that is released in the process. If the cause is determined to be excess bradykinin, a clinician may suspect angiotensin-converting enzyme inhibitors, also known as ACE inhibitors (ACEIs). Typically, the angiotensin-converting enzyme (ACE) is responsible for the degradation of both bradykinin and substance P. When this enzyme is inhibited, the elevated levels of bradykinin increase vasodilation and vascular permeability of the postcapillary venules, resulting in an edematous state. ACE inhibitor-associated angioedema has a predilection for the lips, tongue, face, and upper airway, which may progress to airway obstruction [1]. One study observing the management of ACE inhibitor-associated edema in the ED setting noted that 41% of patients with this subtype of angioedema required admission for inpatient care [28]. Another study shows among patients who presented for emergency treatment, 16% required intubation because of symptoms of drooling and respiratory distress [29]. Used in a variety of indications, including hypertension, heart failure, proteinuria, and diabetic nephropathy, ACEI-induced angioedema affects 0.1-6% of all patients who are taking this medication [27]. The average onset of symptoms after starting ACE inhibitor therapy is reported to be 1.8 years, with 25% of these patients experiencing symptoms within the first month [27]. One risk factor for this bradykinin-associated angioedema is when the ACE’s reinforcements fail. When ACE is inhibited, it is the responsibility of secondary metabolic enzymes to cleave bradykinin and substance P. These include neprilysin, aminopeptidase P, and DPP-IV. Deficiencies in these secondary enzymes are capable of predisposing patients to bradykinin-associated angioedema development when started on an ACE inhibitor [1]. In addition, there is an increased prevalence of this subtype of angioedema in patients of African descent compared to Caucasians. This ethnic variation may, in part, be due to a polymorphism (MME, rs989692) in the genes encoding the enzymes neprilysin and aminopeptidase P, responsible for degrading bradykinin [1]. Other risk factors associated with ACE inhibitor-associated angioedema include smoking history, seasonal allergies, antihistamine use, and corticosteroid use, with studies showing an increased number of presentations during the months with higher pollen counts [1].

The diagnosis of ACE inhibitor-associated angioedema is made clinically based on the presence of angioedema without urticaria and pruritus [27]. Though ACE inhibitor-associated angioedema will appear similar to HAE with bradykinin overactivation, obtaining serum levels of C1 inhibitor and C4 will assist in the diagnosis [27]. If the angioedema is the result of an ACE inhibitor, the patient will have normal levels of C1 inhibitor and C4, effectively ruling out the hereditary subtypes. Discontinuing the ACE inhibitor and monitoring for resolution of the angioedema confirms the diagnosis and is the mainstay of treatment in addition to airway management [1]. Currently, there is conflicting information on the medical management of ACE inhibitor-associated angioedema. A beta2 antagonist, icatibant, has been FDA-approved for use in HAE. In theory, because both HAE and ACE inhibitor-associated angioedema are the results of excess bradykinin, icatibant should also be beneficial in the latter; however, two separate randomized clinical studies have determined there was no effect on the resolution of symptoms when caused by ACE inhibitors [1]. Similarly, another treatment option for HAE that has proven ineffective in ACE inhibitor-associated angioedema is ecallantide, a kallikrein inhibitor. The ineffectiveness of these therapies suggests that there may be an underlying substrate of ACE inhibitors that results in continued symptoms despite the beta2 receptor blockade in these patients [1]. Therefore, the most important step in managing these patients is the discontinuation of the ACE inhibitor. Patients who have experienced this angioedema should avoid all other ACE inhibitors in the future because this adverse effect is class-specific [27]. The next question is whether the patient can safely take an angiotensin receptor blocker (ARB) in place of the ACE inhibitor. In the Telmisartan Randomized Assessment in ACE Intolerant Subjects with Cardiovascular Disease trial, two of 2954 patients receiving telmisartan had angioedema [1]. In comparison, only three of 2972 patients receiving a placebo experienced angioedema, effectively demonstrating that the risk of angioedema in patients using ARBs is similar to the general population [1].

Non-steroidal anti-inflammatory drugs

Another pharmaceutical class that has demonstrated its ability to cause angioedema are NSAIDs, with ibuprofen and aspirin being the most common offender [27]. This angioedema is considered non-allergic and is the result of intolerance, with a prevalence of 0.3 to 0.9% in the average population [27]. The European Academy of Allergy and Clinical Immunology Task Force on NSAID Hypersensitivity has proposed five different categories for immediate-onset NSAID reaction, 3 of which include angioedema in the presentation [1]. The first of these is NSAID-induced angioedema in patients who have no preexisting predisposition to urticaria and angioedema. A patient in this category will experience angioedema only when taking a particular NSAID. No symptoms will present when the patient uses NSAIDs of other chemical structures [1]. The mechanism behind this structure-specific reaction is thought to be due to an IgE-mediated type 1 hypersensitivity reaction, resulting in mast cell degranulation and the release of histamine. For this category, the treatment is simple: discontinuation of NSAIDs that provoke a reaction and avoid all other NSAIDs that are structurally related to the one that caused the angioedema [1]. The second category of NSAID-induced angioedema includes reactions in patients who will have urticaria and angioedema symptoms with any NSAID despite structural differences [27]. This subtype is the result of cyclooxygenase 1 and 2 inhibition, interfering with arachidonic acid metabolism and decreasing the production of prostaglandins [27]. As a result, the pathway is diverted toward the production of leukotrienes B4, C4, and D4, which cause plasma extravasation and vasodilation. Studies have suggested that certain genetic variants predispose patients to this subtype of NSAID-induced angioedema, including ALOX5, ALOX15, CYSLTR1, PTGDR, and PTGER1 [1]. In these patients, acetaminophen is typically safe for use and is the preferred alternative. The third category of NSAID-induced angioedema presents in patients with a history of chronic urticaria and/or angioedema [1]. This is termed NSAID-exacerbated disease because the patient is already predisposed to angioedema, and when combined with the use of NSAIDs, the patient’s symptoms can increase in severity [1]. Similar to the second category, this subtype is also defined by the alteration of arachidonic acid metabolism resulting in an imbalance between leukotrienes and prostaglandins. However, because NSAIDs exacerbate an underlying disease, NSAID desensitization is not an effective form of therapy in these patients. Instead, treatment should include addressing the chronic issue of angioedema [1].

Despite many categories of NSAID-induced angioedema, the clinical features are similar in each subtype. In addition, NSAID-induced angioedema doesn’t differ significantly from allergy-mediated angioedema [27]. Both will present within minutes to a few hours of administration of the NSAID [27]. Because of this, the most reliable test for NSAID intolerance and hypersensitivity is an oral challenge. Testing for histamine and leukotriene release is often expensive, impractical, and isn’t routinely done [1]. In treating NSAID-induced angioedema, acute management in the ED is similar to allergy-induced angioedema, while also warning patients to avoid NSAIDs as a class.

Beta-lactam antibiotics

Beta-lactam antibiotics are bactericidal agents which act by interrupting bacterial wall formation. This is a through covalent binding to essential penicillin-binding proteins (PBPs) [30]. These are enzymes that work in the terminal steps of peptidoglycan cross-linking. PBPs can be divided into different classes according to molecular mass. Low molecular mass PBPs serve in monofunctional carboxypeptidases. High molecular mass PBPs are divided into two subclasses. One includes bifunctional enzymes, and the other includes dependent transpeptidases.

Angioedema is the result of deep dermal swelling, which could be life-threatening, especially in cases where the pharynx and larynx are involved [31]. Drug-induced angioedema is reported to occur from a plethora of drugs and vaccines. Drug-induced angioedema is seen to be most frequently elicited by beta-lactam antibiotics. Beta-lactams are antibiotics that regularly provoke reactions that are controlled by specific immunological mechanisms [30]. The reactions are classified as immediate or non-immediate, and they can be produced by the four classes of beta-lactams. The four classes include penicillins, cephalosporins, carbapenems, and monobactams. These all share a beta-lactam ring structure. Immediate reactions are seen to occur within the first hour of the drug being administered. These reactions include urticaria, angioedema, and bronchospasm. The most reliable method for showing the presence of these beta lactam-specific IgE antibodies is through immediate reading skin tests. If the result is negative, physicians should treat patients with the knowledge that sensitivity will decrease over time. Ultimately, this information means the time elapsed since taking the beta-lactam should be taken into consideration. The management of immediate allergic reactions takes into consideration both the severity and the type.

Beta-lactams can cause several allergic reactions. These are classified as immediate, accelerated, or delayed, and it is classified according to the time interval between the drug administration and the onset of the allergic reaction [32]. These are further classified as immediate or non-immediate reactions. Immediate reactions occur in the first hour of drug administration. This is shown to be urticaria and/or angioedema. Non-immediate reactions occur more than an hour after drug administration. These reactions include maculopapular or morbilliform exanthems. These are seen during treatment with amoxicillin or ampicillin. Beta lactams can also show delayed angioedema and more severe reactions like Stevens-Johnson syndrome. Beta lactams can also cause nephritis, pneumonitis, hepatitis, or vasculitis. Beta-lactam antibiotics have a multitude of side effects and reactions, which can truly alter someone's life and cause serious side effects. These antibiotics are to be taken with caution because of these side effects.

Other drugs

Dipeptidyl Peptidase IV Inhibitors

It has been well studied the comorbid and often deleterious relationship between hypertension and diabetes. The macro and microvascular changes seen in these conditions put patients at higher risk for metabolic derangements and conditions such as cardiovascular disease, neurovascular changes, end-stage renal disease, and peripheral nerve damage [33-35]. First-line treatment for hypertension in patients with diabetes mellitus are inhibitors of the renin-angiotensin-aldosterone system, particularly ACEIs, and aldosterone receptor blockers, partially for its renal protective effects [34]. Additionally, for the treatment of hyperglycemia in type 2 diabetes mellitus DPP-IV inhibitors, a class of drugs known as -gliptins, have been utilized.

DPP-IV inhibitors, a class of drugs known as -gliptins, have been utilized for the treatment of type 2 diabetes mellitus. DPP-IV is an enzyme that quickly degrades several incretin hormones that function to regulate both insulin and glucagon secretions as well as gastric emptying. These enzymes include glucagon-like peptide (GLP-1) and gastric inhibitory peptide (GIP) [36]. Inhibiting the breakdown of GLP-1 and GIP leads to their prolonged effects of delayed gastric emptying and insulin secretion, lending to its beneficial effects in hyperglycemic control. Additionally, DPP-IV has been shown to degrade other peptides such as bradykinin and substance P; however, at much lower levels than other enzymes such as ACEs that are the primary metabolic pathway of degradation [37,38]. Increased levels of bradykinin have been implicated in drug-induced angioedema via a proposed mechanism of increased vascular permeability induced by bradykinin 2 receptor activation. Saxagliptin and sitagliptin, both FDA-approved DPP-IV inhibitors, made updates to medication package inserts following post-marketing reports of angioedema [39,40]. Several case reports have been documented of patients taking both ACE inhibitors and DPP-IV inhibitors experiencing bradykinin-induced angioedema; however, no single gliptin had been implicated across case reports.

A meta-analysis was conducted by Brown et al., examining the FDA Adverse Events Reports prior to 2007 for several gliptins, including vildagliptin, sitagliptin, saxagliptin, and alogliptin. Of the 13,921 cases reviewed, an independent internal medicine adjudication committee confirmed 27 cases of angioedema. Of the 27 confirmed cases, 19 were taking vildagliptin, and of those, 14 patients were also taking an ACE inhibitor concurrently [37]. As mentioned previously, ACE is the primary metabolic pathway for the degradation of bradykinin, and when ACE levels are diminished, such as in patients taking ACE inhibitors, the role of DPP-IV in vasoactive peptide degradation increases. Concomitant use of ACE inhibitors and the DPP-IV inhibitor vildagliptin in the Brown et al. meta-analysis showed that patients had an increased risk of angioedema with an odds ratio of 4.57 (95% confidence interval 1.57 to 13.28) [37]. Though the limitation of this study includes the few incidences of angioedema and selection bias for patients who have a lower risk of ACE inhibitor-induced angioedema (a common side effect of ACE inhibitor), this increased risk is something to be cautious of when prescribing both ACE inhibitors and DPP-IV inhibitors.

A study was completed to assess saxagliptin and cardiovascular outcomes in patients with type 2 diabetes. Though angioedema was not a primary endpoint and the definition of "angioedema event" was not defined in the study protocols, it was noted that in the saxagliptin group (N=8280) and the placebo group (N=8218), there were eight cases of non-fatal angioedema vs one in the placebo group [41]. A disproportionality analysis of VigiBase was conducted in 2020, and findings indicated that concomitant use of ACE inhibitors and DPP-IV inhibitors as a class, not a singular gliptin, had increased reports of bradykinin-mediated angioedema. About 345 reports were reviewed meeting the criteria of bradykinin-mediated angioedema with concomitant ACE inhibitor and DPP-IV inhibitor use. There were several gliptins reported, including 225 sitagliptin, 43 saxagliptin, 40 vildagliptin, 37 linagliptin, and 3 alogliptin [42]. An additional pharmacovigilance study was conducted examining FDA Adverse Events Report data from 2013 to 2017 and noted that linagliptin had an association with angioedema irrespective of ACE inhibitor use [43].

Neprilysin Inhibitors

Neprilysin is part of a family of zinc metallopeptidase that is ubiquitous in its distribution throughout the body and peptide substrates for degradation [9,44-46]. These substrates include vasoactive peptides atrial natriuretic peptide (ANP), B-type natriuretic peptide (BNP), C-type natriuretic peptide (CNP), bradykinin, substance P, adrenomedullin, vasoconstrictive peptides endothelin 1 and Angiotensin II, amyloid beta, GLP-1, vasoactive intestinal peptide, and insulin-B chains [45,46]. Neprilysin's affinity for ANP, BNP, and angiotensin II makes its inhibition a promising target for utilization in the management of cardiovascular diseases such as hypertension and heart failure. However, the reduced degradation of bradykinin with neprilysin inhibition has shown an increased incidence of angioedema events.

Omapatrilat, a combined neprilysin inhibitor, and ACEI were investigated in a multicenter, randomized, double-blind active control trial in comparison to enalapril as an antihypertensive. At the conclusion of the 24-week study, omapatrilat had a statistically significant reduction of systolic blood pressure, reduced use of additional antihypertensives, and an increase in the number of participants achieving target blood pressure compared to the enalapril subject group [47]. Unfortunately, the increased incidence and severity of angioedema-related events induced by drug treatment led to a reduction in efforts for continued production [45,48]. In the omapatrilat-treated group, 2.17% of subjects had an angioedema event (two of which required airway management) compared to 0.86% in the enalapril group. Additionally, the omapatrilat treatment group had an increased severity of angioedema events, African Americans in this treatment group experienced a three-fold increase in angioedema, and current smokers in the omapatrilat treatment group had an increased incidence of angioedema of 3.93% [47].

The Prospective Comparison of angiotensin receptor-neprilysin inhibitor with ACEI to determine the impact on Global Mortality and Morbidity in Heart Failure Trial (PARADIGM-HF) study assessed the combination drug ARB valsartan with neprilysin inhibitor sacubitril (LCZ 696) versus enalapril which showed significantly reduced mortality in patients with heart failure with reduced ejection fraction with a lower incidence of angioedema events compared to omapatrilat [48]. Authors note that this reduction in angioedema events compared to neprilysin inhibitor and ACE inhibitor combination may be due to ARB decreased degradation of ACE and aminopeptidase P compared to ACE inhibitors. Both ACE and aminopeptidase P are utilized in the degradation of bradykinin. Though the incidence of angioedema did not rise to the level of statistical significance, and no incidence led to airway compromise in either group, there was still an increase in the number of angioedema events in the LCZ696 treatment group (19 of N=4187) compared to enalapril-group (10 of N=4212) [48,49].

Tissue Plasminogen Activator

tPA and recombinant tissue plasminogen activator (rtPA or alteplase) are utilized for the acute management of ischemic cerebral strokes as well as other thrombotic events such as myocardial infarctions and deep vein thrombosis. tPA hydrolyzes plasminogen to plasmin and, in the process, produces vasoactive peptides such as bradykinin [50]. Increased bradykinin is thought to activate the bradykinin 2 receptors leading to angioedema.

A retrospective analysis was conducted at a United Kingdom medical center to assess the incidence of orolingual angioedema in patients receiving tPA between 2004 and 2012. About 530 patients were included in the final study cohort of individuals who had suffered an ischemic stroke and received tPA. Of note, 33% (172 patients) were taking ACE inhibitors. Angioedema occurred in 7.9% (95% CI 5.5% to 10.6%). With an odds ratio of 2.3, a significant predictor of angioedema was previous patient use of ACE inhibitors (95% CI 1.1 to 4.7; p=0.02) [51].

**Figure 1:**
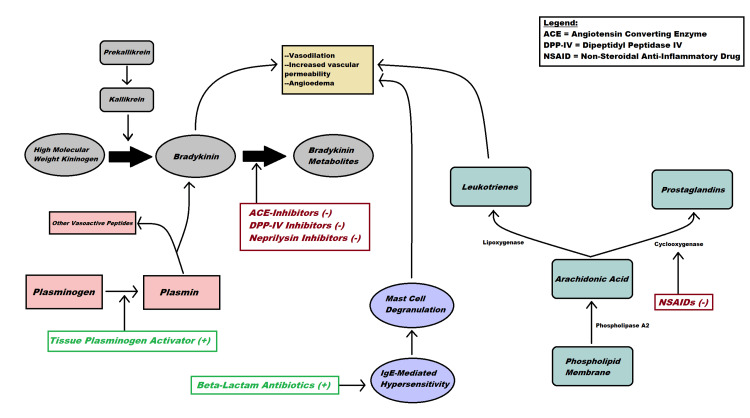
Mechanisms of pathogenesis of drug-induced angioedema

Additionally, studies conducted with bradykinin 2 receptor knockout mice concluded that after induced middle cerebral artery occlusion, there were increased levels of bradykinin seen in brain parenchyma. Messenger ribonucleic acid (mRNA) for the bradykinin 2 receptor was increased in neurons showing signs of ischemia [52]. Both these phenomena could be implicated in the increased incidence of angioedema following ischemic stroke in individuals receiving tPA (Figure 1 and Table 1).

**Table 1:** Drugs that can commonly cause angioedema ACE: Angiotensin-converting enzyme; ARB: Angiotensin receptor blocker; NSAID: Non-steroidal anti-inflammatory drug; DPP-4: Dipeptidyl peptidase-4, tPA: Tissue plasminogen activator Reference: [1,6,31,53]

Class of medication	Mechanism of action
Angiotensin-converting enzyme inhibitors	Inhibition of ACE results in decreased degradation of bradykinin, a potent vasodilator and permeability factor, leading to increased levels of bradykinin and subsequent angioedema.
Angiotensin receptor blockers	Similar to ACE inhibitors, ARBs can result in increased bradykinin levels and subsequent angioedema, though less frequently than inhibitors.
Non-steroidal anti-inflammatory drugs	Inhibition of cyclooxygenase enzymes results in decreased production of prostaglandins, which normally inhibit histamine release. Histamine release then leads to angioedema, though NSAIDs can also cause angioedema through other mechanisms, such as activation of the kinin system.
Antibiotics (penicillins, sulfonamides, cephalosporins)	Antibiotics can cause angioedema through both immune-mediated and non-immune-mediated mechanisms. Immune-mediated angioedema can result from the activation of the complement system, leading to the generation of anaphylatoxins such as C3a and C5a, which can stimulate the release of histamine from mast cells. Non-immune-mediated angioedema can result from direct activation of mast cells by the antibiotic.
Dipeptidyl peptidase-4 inhibitors	DPP-4 inhibitors can increase levels of bradykinin by inhibiting its breakdown, leading to increased levels of bradykinin and subsequent angioedema.
Neprilysin inhibitors	Neprilysin inhibitors can increase levels of vasoactive peptides such as bradykinin and substance P, leading to increased levels of bradykinin and subsequent angioedema.
Tissue plasminogen activator	tPA can activate the complement system and induce the release of vasoactive peptides such as bradykinin, leading to increased levels of bradykinin and subsequent angioedema.

## Conclusions

Angioedema is a localized swelling of the dermis, submucosal tissues, and subcutaneous tissues, which is caused by fluid buildup in these tissues. This topic is important because of the number of people affected by this, and it is important to summarize the causes and reasons for why this occurs. Drug-induced angioedema occurs from mechanisms that are mediated by an accumulation of vasoactive molecules such as histamine, leukotriene, and bradykinin. Angioedema is responsible for 80,000-112,000 ED visits every year. The hospitalization rate is four per 100,000. This affects many people and has serious side effects. Patients who show up with angioedema usually involve edema of the lips, tongue, and face. It can more seriously affect the larynx, as well. The involvement of the gastrointestinal tract occurs in patients who have HAE. Management of angioedema includes removing the agent that is causing the symptoms. Multiple drugs can cause angioedema, including beta-lactam drugs which is the drug that most commonly provokes reactions such as angioedema. Genetic factors include a personal history of allergies or use of antihistamines, which have an increased risk of ACEI-induced angioedema. Overall, angioedema is a life-threatening issue, and multiple drugs and other risks can cause it. Additional studies related to understanding the mechanisms of angioedema and therapeutic interventions are warranted in the future.
